# Anti-Inflammatory Actions of Plant-Derived Compounds and Prevention of Chronic Diseases: From Molecular Mechanisms to Applications

**DOI:** 10.3390/ijms26115206

**Published:** 2025-05-28

**Authors:** Kazuhiko Nakadate, Nozomi Ito, Kiyoharu Kawakami, Noriko Yamazaki

**Affiliations:** 1 Department of Functional Morphology, Meiji Pharmaceutical University, 2-522-1, Noshio, Kiyose 204-8588, Tokyo, Japan; m256206@std.my-pharm.ac.jp (N.I.); k-kawakami@my-pharm.ac.jp (K.K.); 2 Department of Community Health Care and Sciences, Meiji Pharmaceutical University, 2-522-1, Noshio, Kiyose 204-8588, Tokyo, Japan; nyamazak@my-pharm.ac.jp

**Keywords:** inflammatory, chronic diseases, cardiovascular diseases, type 2 diabetes, neurodegenerative disorders, cancer, polyphenols, flavonoids, carotenoids, obesity

## Abstract

Chronic inflammation is a key contributor to the development and progression of numerous chronic diseases, including cardiovascular diseases, type 2 diabetes, neurodegenerative disorders, cancer, and obesity. As the side effects of conventional anti-inflammatory drugs pose challenges, plant-derived compounds have emerged as promising alternatives due to their potent anti-inflammatory properties and minimal adverse effects. This review explores the molecular mechanisms by which these compounds alleviate chronic inflammation and highlights their potential role in disease prevention. Polyphenols (e.g., quercetin and resveratrol), flavonoids (e.g., luteolin and apigenin), carotenoids (e.g., β-carotene and lycopene), and other phytochemicals (e.g., curcumin and gingerol) modulate inflammatory pathways, such as nuclear factor-κB and mitogen-activated protein kinase, reduce oxidative stress, and inhibit pro-inflammatory cytokines. Plant-derived compounds interact with the gut microbiota, enhancing anti-inflammatory effects. Evidence from animal studies and clinical trials has demonstrated their efficacy in reducing inflammation-related biomarkers and improving health outcomes. However, challenges such as low bioavailability and determination of the optimal dosage require further investigation. Advancing delivery technologies and personalized nutrition strategies may help overcome these barriers. This review emphasizes the therapeutic potential of plant-derived compounds in preventing chronic diseases and underscores the need for continued research to translate these findings into practical applications for public health.

## 1. Introduction

Chronic inflammation is considered one of the most important and complex health challenges in modern medicine [1,2]. Chronic inflammation is a low-grade inflammatory condition caused by the body’s exposure to persistent irritation and stress, which, when sustained over time, promotes the progression of many major chronic diseases, including cardiovascular disease (CVD), metabolic syndromes, type 2 diabetes, Alzheimer’s disease, Parkinson’s disease, and cancer [3,4,5]. Behind this chronic inflammation lies a persistent over-activation of the immune response, persistent overproduction of inflammatory cytokines (interleukin-1β [IL-1β], interleukin-6 [IL-6], tumor necrosis factor-α [TNF-α], etc.), overproduction of reactive oxygen species (ROS), and associated cellular damage and tissue dysfunction [6,7,8]. These factors interact to form a vicious cycle of inflammation that accelerates disease onset and progression.

For example, in CVDs, chronic inflammation causes dysfunction of vascular endothelial cells and promotes the progression of atherosclerosis [9,10]. Furthermore, inflammatory cytokines have been shown to disrupt blood pressure regulation and induce vascular wall remodeling [11,12]. On the other hand, in metabolic syndromes, type 2 diabetes, and chronic obesity, chronic adipose tissue and hepatic inflammation exacerbate insulin resistance and disrupt glucose metabolism [13,14,15]. In neurodegenerative diseases, chronic inflammation in the brain leads to neuronal damage and apoptosis and is deeply involved in the progression of Alzheimer’s disease and Parkinson’s disease [16]. In cancer, chronic inflammation has been reported to alter the tumor microenvironment and promote cancer cell growth and metastasis [17,18]. Chronic inflammation triggers these diseases and is a factor in disease progression.

**Figure 1:**
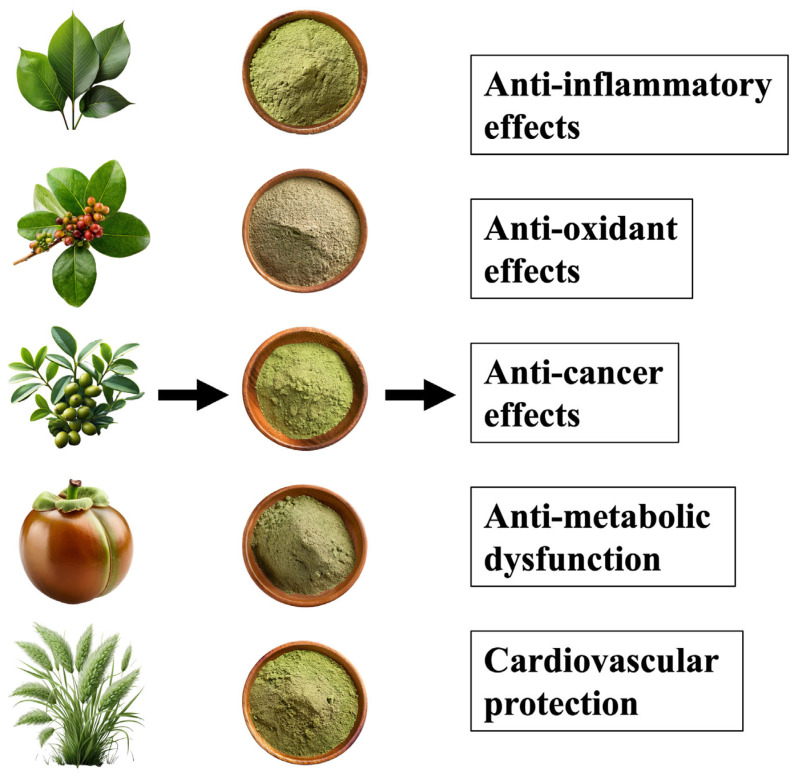
Plant-derived bioactive compounds improve our health.

In modern medicine, chemical therapies and pharmaceuticals are used to control chronic inflammation [19]. For example, nonsteroidal anti-inflammatory drugs (NSAIDs) and steroids are widely used to control inflammation because they inhibit the production of inflammatory cytokines [20,21]. However, these therapies are associated with side effects and risks with long-term use; NSAIDs can cause gastrointestinal disturbances and impaired renal function, whereas steroids cause immunosuppression and metabolic abnormalities [22,23]. Therefore, new approaches are needed to control inflammation safely and effectively. Against this background, plant-derived compounds have attracted attention (Figure 1). These compounds are extracted from naturally occurring foods, medicinal herbs, and spices and are expected to have diverse health effects, including anti-inflammatory, antioxidant, immunomodulatory, and even cancer prevention effects. In particular, polyphenols (e.g., quercetin and resveratrol), flavonoids (e.g., luteolin and apigenin), carotenoids (e.g., β-carotene and lycopene), and other plant compounds (e.g., curcumin and gingerol) have proven their anti-inflammatory effects in numerous studies [24,25,26,27,28,29].

The molecular mechanisms by which plant-derived compounds exert their anti-inflammatory effects are diverse [30,31]. These compounds not only suppress the activity of key inflammatory signaling pathways, such as nuclear factor-κB (NF-κB) and mitogen-activated protein kinase (MAPK), but also reduce oxidative stress and prevent cell damage by decreasing ROS [32,33]. They have also been shown to disrupt the vicious cycle of inflammation by suppressing the production of inflammatory cytokines [34]. Furthermore, through their interaction with intestinal microflora, it has been suggested that they may reduce systemic inflammation [35]. Therefore, plant-derived compounds are considered promising tools in the prevention and management of chronic diseases.

This study provides a comprehensive review of how plant-derived compounds contribute to the prevention of chronic inflammation and related diseases. Specifically, the molecular mechanisms underlying the anti-inflammatory effects of these compounds, results from disease models and clinical studies, and the challenges and prospects for their practical application are detailed. The potential role of plant-derived compounds in medicine and public health is discussed, and their potential for the next generation of inflammation therapy and disease prevention is explored.

## 2. Materials and Methods

For this review, literature searches were conducted using the electronic databases PubMed, ScienceDirect, and Web of Science, with searches focusing on recent data. Search terms included the following: “plant-derived compound”, “inflammatory”, “oxidative stress”, “chronic disease”, “neurodegenerative disease”, “cancer”, polyphenol”, “flavonoid”, “carotenoid”, “curcumin”, and “gingerol”. Duplicate references were removed. Based on this search and criteria, the cited articles were refined for this review.

## 3. Chronic Inflammation and Disease Relationships

### 3.1. Mechanisms of Chronic Inflammation

Chronic inflammation is caused by repeated or prolonged exposure to internal and external stressors. These factors include infections, persistent trauma, lifestyle habits such as obesity and inactivity, and environmental factors such as smoking and air pollution [36,37,38,39,40]. These factors can lead to an uncontrolled immune response and cause the body to undergo the following characteristic processes.

#### 3.1.1. Overproduction of Inflammatory Cytokines

Inflammatory cytokines (such as IL-1β, IL-6, and TNF-α) are important molecules produced by the immune system to enhance inflammatory responses [41,42,43]. In a normal immune response, these molecules play an important role in repairing the site of infection or injury, but in conditions of chronic inflammation, they are overproduced and attack healthy cells and tissues. As a result, local tissue damage leads to systemic pathological changes.

#### 3.1.2. Oxidative Stress and ROS Generation

During the inflammatory process, immune cells (especially neutrophils and macrophages) produce ROS [44,45]. While ROS attacks pathogens, excessive ROS accumulation damages cell membranes, proteins, and DNA, and DNA damage can lead to cell death and mutations, contributing to an increased risk of cancer [46]. In addition, oxidative stress further activates chronic inflammatory pathways, creating a vicious cycle of inflammation [47].

#### 3.1.3. Activation of Inflammatory Signaling Pathways

In states of chronic inflammation, key intracellular signaling pathways are homeostatically activated. In particular, NF-κB, MAPK, and Janus kinase/signaling and transcription activator pathways are involved [48,49]. These pathways induce the expression of inflammatory cytokines, chemokines, prostaglandins, and other inflammatory molecules [50,51,52]. This process contributes significantly to the persistence of pathologic inflammation.

#### 3.1.4. Immune Cell Hyperactivation and Tissue Infiltration

In chronic inflammatory conditions, immune cells (macrophages, microglia, neutrophils, etc.) are over-activated and persistent infiltration of the inflammatory site is observed [53]. These immune cells produce additional inflammatory mediators that also damage surrounding healthy tissue [54]. As this process progresses, inflammation becomes the primary cause of autoimmune and chronic diseases.

#### 3.1.5. Polyphenol-Mediated Regulation of Antioxidant Enzymes: Focus on Catalase

Polyphenols exert strong antioxidant effects by directly scavenging ROS and modulating the expression and activity of endogenous antioxidant enzymes, such as catalase, superoxide dismutase, and glutathione peroxidase [55,56]. Among these, catalase plays a crucial role in the detoxification of hydrogen peroxide (H_2_O_2_), a major ROS involved in oxidative stress and chronic inflammation.

Several studies have demonstrated that polyphenols, including resveratrol, quercetin, epigallocatechin gallate (EGCG), and curcumin, can enhance catalase activity through transcriptional and post-translational mechanisms [57,58]. For example, resveratrol has been shown to upregulate catalase gene expression via the activation of the Nrf2 signaling pathway, a master regulator of antioxidant response [59]. Similarly, quercetin increases catalase activity in hepatic and endothelial cells by promoting Nrf2 nuclear translocation and binding to the antioxidant response element (ARE) in the catalase promoter region [60].

In addition to gene regulation, polyphenols may stabilize catalase protein or protect it from oxidative inactivation. For instance, EGCG and curcumin have been reported to preserve catalase activity under oxidative stress conditions in models of neurodegeneration and metabolic syndrome [60]. These polyphenol-mediated effects on catalase contribute significantly to reductions in cellular ROS levels, maintenance of redox homeostasis, and the prevention of ROS-induced damage in various chronic diseases.

### 3.2. Role of Inflammation in Chronic Disease

Chronic inflammation is involved in the development and progression of nearly all major chronic diseases, and the mechanisms involved differ for each disease. Chronic inflammation is closely associated with the following: CVDs, metabolic syndromes, type 2 diabetes, neurodegenerative diseases, and chronic obesity. The role of chronic inflammation in these diseases will be discussed in detail.

#### 3.2.1. CVDs

Chronic inflammation is one of the major pathological mechanisms in CVDs, such as atherosclerosis and hypertension [61]. Inflammatory cytokines (especially IL-6 and TNF-α) impair vascular endothelial cell function and increase endothelial cell permeability [62]. In the process, low-density lipoproteins accumulate in the vessel wall and are oxidized, leading to the formation of atherosclerotic plaques [63]. Chronic inflammation also accelerates the progression of atherosclerotic disease by causing a breakdown in blood pressure regulation and promoting vessel wall remodeling [64].

#### 3.2.2. Metabolic Syndromes and Type 2 Diabetes

Chronic inflammation is directly involved in the development and exacerbation of insulin resistance [65]. With obesity, inflammatory cytokines (especially TNF-α and IL-6) secreted by adipose tissue inhibit insulin receptor signaling and cause chronic elevations in blood glucose levels. In addition, adipokine secretion from adipocytes is abnormal in obesity, amplifying chronic systemic inflammation [66]. The result is a progressive disruption of glucose metabolism and an increased risk of diabetic complications. Recent studies have highlighted the role of semaphorin 3E (Sema3E), a member of the semaphorin family originally known for its function in axon guidance, in mediating adipose tissue inflammation and insulin resistance. Sema3E is highly expressed in the adipose tissue of obese mice and humans, particularly by pro-inflammatory macrophages. This protein binds to its receptor PlexinD1, which is also upregulated in inflamed adipose tissues, leading to enhanced macrophage infiltration and reduced egress from adipose depots. This retention of M1-like macrophages sustains local inflammation and increases the production of cytokines such as IL-6 and TNF-α, which further impairs insulin signaling in adipocytes. Moreover, genetic or pharmacological inhibition of Sema3E/PlexinD1 signaling has been shown to attenuate adipose inflammation, improve insulin sensitivity, and normalize glucose tolerance in obese mice, indicating that Sema3E is not only a marker of metabolic inflammation but is also a functional contributor to insulin resistance. These findings suggest that targeting the Sema3E–PlexinD1 axis may provide a novel therapeutic strategy for mitigating inflammation-induced metabolic disorders.

#### 3.2.3. Neurodegenerative Diseases

Brain inflammation is a major factor in the progression of neurodegenerative diseases such as Alzheimer’s disease and Parkinson’s disease [67]. Microglia, immune cells of the central nervous system (CNS), are chronically activated by pathological stimuli (e.g., amyloid-β accumulation and abnormal alpha-synuclein), releasing inflammatory cytokines and ROS [68]. This inflammatory response leads to neuronal damage and apoptosis and promotes a decline in neurological function [68]. The interaction between brain and vascular inflammation has also been noted to play an important role in the progression of neurodegenerative diseases.

#### 3.2.4. Cancer

Chronic inflammation is a potent promoter of cancer development and progression [69]. Persistent chronic inflammation leads to the secretion of inflammatory cytokines and growth factors (e.g., IL-6, VEGF), which modify the tumor microenvironment [70]. This environment promotes cancer cell proliferation, angiogenesis, and metastasis and protects cancer cells from the immune system. In addition, inflammation-induced oxidative stress and DNA damage can induce mutations and increase carcinogenicity [71,72].

Moreover, the epidermal growth factor receptor (EGFR) signaling pathway plays a critical role in linking chronic inflammation to tumorigenesis. EGFR is frequently overexpressed or mutated in various cancers and is activated by inflammatory mediators such as TNF-α and IL-1β [73]. EGFR activation stimulates downstream signaling cascades, including the RAS-RAF-MEK-ERK and PI3K-AKT pathways, which promote cell proliferation, survival, and resistance to apoptosis [74]. Inflammatory conditions can enhance EGFR signaling directly through ligand induction (e.g., EGF, TGF-α) and indirectly via ROS-mediated receptor activation, further contributing to an environment that is conducive to tumor initiation and progression [75]. Notably, plant-derived compounds such as curcumin, resveratrol, and EGCG have been shown to inhibit EGFR signaling and its downstream pathways, exerting anti-proliferative and anti-inflammatory effects in cancer models [76].

#### 3.2.5. Chronic Obesity

Chronic obesity is characterized by persistent low-grade inflammation, and adipose tissue is known to be the primary starting point of inflammation [77]. As obesity progresses, adipose tissue increases the secretion of inflammatory cytokines and promotes macrophage infiltration, triggering a systemic inflammatory response [78]. This chronic inflammation has been reported to exacerbate insulin resistance and metabolic abnormalities, increasing the risk of type 2 diabetes, CVD, and even cancer and neurodegenerative diseases [79].

Enlarged adipocytes secrete excessive amounts of the inflammatory cytokines (TNF-α, IL-6, and IL-1β) [80,81,82]. These cytokines activate immune cells in adipose tissue and cause them to release more inflammatory mediators, forming a vicious cycle of chronic inflammation. TNF-α, in particular, is known to inhibit insulin signaling and cause abnormal glucose metabolism. IL-6 has been reported to promote the production of C-reactive protein in the liver, which is reflected in the blood as a systemic inflammatory marker [83]. With obesity, adipose tissue becomes infiltrated by macrophages, whose phenotype shifts from anti-inflammatory type M2 to inflammatory type M1 [84]. Type M1 macrophages produce large amounts of inflammatory cytokines, further amplifying adipose tissue inflammation. This accumulation of inflammatory macrophages plays an important role in the pathogenesis of obesity, contributing to adipocyte dysfunction and insulin resistance. Furthermore, macrophage-derived nitric oxide and ROS have been shown to increase adipocyte stress responses and induce apoptosis [85,86]. Chronic inflammation associated with obesity inhibits phosphorylation of insulin receptor substrate-1, an important component of the insulin signaling pathway, thereby reducing insulin action. TNF-α has been shown to decrease insulin receptor substrate-1 activity, particularly via serine/threonine phosphorylation, resulting in impaired glucose uptake and regulation of glucose metabolism [87,88]. This exacerbation of insulin resistance is directly involved in the development of type 2 diabetes and contributes to hyperglycemia and dyslipidemia.

Recent studies suggest that alterations in the gut microbiota are an important factor in obesity-related inflammation [89]. Intestinal permeability is increased in the gut of patients with obesity, facilitating the influx of bacteria-derived endotoxins called lipopolysaccharides into the bloodstream. Lipopolysaccharides trigger an inflammatory response via Toll-like receptor 4 and promote the secretion of pro-inflammatory cytokines in the liver and adipose tissue [90,91]. This is known to maintain chronic inflammation in adipose tissue and exacerbate insulin resistance. It has also been suggested that reduced diversity of the intestinal microbiota leads to decreased short-chain fatty acid production and impaired intestinal barrier function, further contributing to inflammation [92].

#### 3.2.6. Dermatological Diseases

Plant-derived compounds have attracted attention as promising natural ingredients for the prevention and treatment of skin diseases due to their diverse biological activities, including anti-inflammatory, antioxidant, and antibacterial effects. Phytoconstituents such as flavonoids, carotenoids, polyphenols, and terpenoids have been reported to have multifaceted effects on the major pathological conditions of skin diseases, such as skin inflammation, oxidative stress, and microbial infection [93,94].

For example, quercetin, luteolin, and apigenin, which are flavonoids, have been shown to be effective in alleviating inflammatory skin diseases such as atopic dermatitis and psoriasis by inhibiting the production of inflammatory cytokines such as TNF-α and IL-6 [95,96]. Quercetin contributes to the reduction of erythema and itching by inhibiting inflammatory signaling [96]. Carotenoids such as lycopene, lutein, and β-carotene have antioxidant properties, which include scavenging UV-induced ROS, and are useful in preventing photoaging and sun-induced skin damage. Oral lutein supplementation has been clinically shown to improve skin hydration and elasticity [97]. Polyphenols (e.g., resveratrol, EGCG, and chlorogenic acid) contribute to the control of acne and skin infections by enhancing skin barrier function and antibacterial activity. In particular, EGCG from green tea has been shown to inhibit sebum secretion and the growth of Cutibacterium acnes, suggesting its potential as a therapeutic aid for acne [94]. Terpenoids (curcumin, boswellic acid, cinnamaldehyde, and so on) exhibit anti-inflammatory effects as well as promote wound healing and antifungal activity. This has led to potential applications in contact dermatitis and fungal skin diseases. In addition, plant extracts and essential oils such as aloe vera, tea tree oil, calendula, and lavender oil are widely used to manage minor burns, itching, and irritating dermatitis due to their moisturizing, antibacterial, and soothing effects.

However, natural ingredients may cause allergic reactions or contact dermatitis in some individuals. Thus, it is important to evaluate safety and tolerability. In addition, challenges exist in the dermal application of plant-derived compounds, such as variability of ingredients, limitations in transdermal absorption, and lack of clinical evidence. To overcome these challenges, recent efforts have been made to improve skin penetration using nanocarrier technology and to develop standardized formulations.

## 4. Major Plant-Derived Compounds and Their Anti-Inflammatory Effects

As shown in Table 1, many plant-derived compounds are gaining attention in the prevention and treatment of chronic diseases because of their naturally occurring yet potent anti-inflammatory properties. These compounds exert their effects through the modulation of inflammatory cytokines, reduction in oxidative stress, and even modification of the immune response. Typical plant-derived compounds include polyphenols, flavonoids, carotenoids, terpenoids, and certain plant-derived molecules such as curcumin and gingerol.

### 4.1. Polyphenols

**Figure 2:**
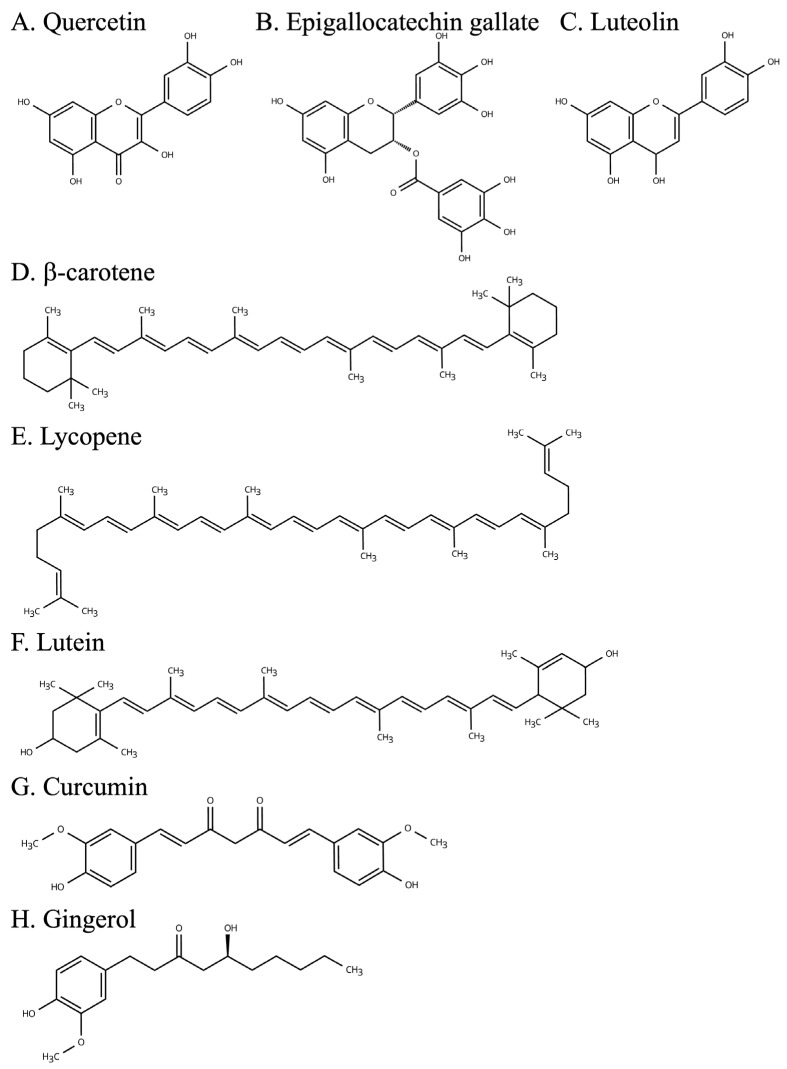
The main plant-derived bioactive compounds discussed in this paper.

Polyphenols are secondary metabolites widely found in the plant kingdom and are known for their antioxidant and anti-inflammatory properties [98,99]. There are many different types of polyphenols, including flavonoids, phenolic acids, stilbenes, and lignans, among which the following examples are of particular interest:

As shown in Figure 2A, Quercetin is a type of flavonoid found mainly in apples and onions, with strong antioxidant and anti-inflammatory properties [100]. Resveratrol, on the other hand, is a stilbene compound found in red wine and grape skins and has been reported to have cardiovascular-protective and anti-inflammatory effects [101]. Furthermore, epigallocatechin gallate, which is found in green tea, has one of the strongest antioxidant activities among catechins and has been shown to inhibit inflammation [102]. These plant-derived compounds are expected to regulate inflammation through different mechanisms of action and contribute to reducing the risk of chronic diseases.

Polyphenols regulate inflammation through several key molecular mechanisms to exert their potent anti-inflammatory effects. First, polyphenols inhibit the activity of NF-κB [103]. NF-κB is a major transcription factor that regulates the expression of inflammatory cytokines, and suppression of its activation prevents an exaggerated inflammatory response. Second, polyphenols act as ROS scavengers and reduce intracellular oxidative stress [104]. Oxidative stress is an aggravating factor in inflammation, and its suppression plays an important role in the control of chronic inflammation. In addition, polyphenols reduce the progression of inflammation by inhibiting the production of inflammatory cytokines such as IL-1β, IL-6, and TNF-α [105]. Through these actions, polyphenols are expected to contribute to the suppression of chronic inflammation and the prevention of associated chronic diseases.

The anti-inflammatory effects of polyphenols have been demonstrated in various disease models and may contribute to preventing or inhibiting the progression of diseases related to chronic inflammation. For example, resveratrol has been shown to reduce the risk of CVD in animal models of the disease [106]. In particular, in atherosclerosis models, resveratrol was shown to reduce inflammation in vascular endothelial cells and prevent atherogenesis. This suggests that it may reduce the onset and progression of CVD. Quercetin has also been reported to reduce intestinal tissue damage in inflammatory bowel disease models by decreasing the expression of the inflammatory enzyme cyclooxygenase-2 [107]. This may improve symptoms and prevent inflammatory bowel disease. Furthermore, epigallocatechin gallate derived from green tea has been shown to suppress the production of inflammatory mediators secreted by fat cells in studies that involved obese mouse models (Figure 2B) [82]. This action has been suggested to reduce chronic inflammation, leading to a lower risk of diabetes through improved insulin resistance. These results indicate that polyphenols are promising candidates for the prevention and treatment of inflammatory diseases and warrant further study.

Flavonoids are a type of polyphenol, a group of compounds that give plants their pigments and bitter taste. Recent studies have revealed that flavonoids are more than just antioxidants; they selectively inhibit specific inflammatory pathways, thereby reducing chronic inflammation and likely contributing to the prevention and treatment of various diseases [108]. As a result, flavonoids are attracting attention as a new therapeutic strategy for inflammatory diseases. Luteolin and apigenin are representative flavonoids [109,110]. As shown in Figure 2C, Luteolin is abundant in celery and bell peppers and is known for its powerful antioxidant and anti-inflammatory properties [109]. Apigenin, on the other hand, is abundant in parsley and chamomile and has been reported to have anti-tumor and anti-inflammatory effects [110]. These flavonoids can inhibit inflammation and reduce disease progression through specific molecular mechanisms.

Flavonoids are known to inhibit the activity of the inflammatory enzyme cyclooxygenase-2 and induce nitric oxide synthase [111]. Induced nitric oxide synthase causes cellular damage by producing excessive nitric oxide. Excessive activity of these enzymes results in a persistent chronic inflammatory state, leading to tissue damage and disease progression. By decreasing the expression and activity of these enzymes, flavonoids may control the progression of inflammation and reduce the risk of developing chronic diseases [108]. Flavonoids are known to inhibit MAPK and NF-κB pathways, which are major signal transduction pathways in inflammation [112]. The MAPK pathway regulates the inflammatory response of cells to external stimuli and stress, and the NF-κB pathway is an important transcription factor that promotes the expression of inflammatory cytokines (IL-1β, IL-6, TNF-α, etc.) [113]. By inhibiting these pathways, flavonoids are considered to suppress inflammatory responses and prevent the vicious cycle of chronic inflammation. In particular, luteolin and apigenin have been reported to show high inhibitory activity against these pathways and effectively suppress inflammation [114].

It has been suggested that flavonoids may interact with the gut microbiota and potentiate their anti-inflammatory effects [115]. Gut bacteria are known to metabolize flavonoids and produce bioactive metabolites, and this metabolic process enhances the anti-inflammatory effects of flavonoids. Flavonoids may also reduce systemic inflammation levels by improving the balance of the gut microbiota and strengthening the gut barrier function [116]. Since intestinal bacterial imbalance plays a role in the progression of chronic diseases such as inflammatory bowel disease and metabolic syndrome, the effects of flavonoids on the intestinal environment are important from a disease prevention perspective.

### 4.2. Carotenoids

Carotenoids are fat-soluble pigments widely distributed in plants and algae that play an important role in maintaining human health. In particular, they are noted for their ability to protect cells from oxidative stress and reduce the risk of developing inflammatory diseases through their potent antioxidant properties [117]. Carotenoids are consumed in food and are known to provide various health benefits. Typical carotenoids include β-carotene (Figure 2D), lycopene (Figure 2E), and lutein (Figure 2F). β-carotene is abundant in carrots and squash and functions as a precursor to vitamin A in the body. Lycopene is abundant in tomatoes and watermelon and is known for its strong antioxidant properties, especially among carotenoids. Lutein, on the other hand, is found in spinach and kale and contributes primarily to the maintenance of eye health; it has also been reported to protect against inflammatory diseases.

Carotenoids protect cells from oxidative stress by inhibiting the oxidation (lipid peroxidation) of lipids that make up cell membranes [118]. Progressive lipid peroxidation leads to cell membrane damage and the activation of inflammatory responses, which increases the risk of developing chronic diseases [119]. Carotenoids help maintain tissue health by directly removing ROS and preventing lipid oxidation. Carotenoids reduce cellular oxidative stress by removing harmful ROS such as superoxide (O_2_-) and hydroxyl radicals (OH-) [120]. This prevents DNA and protein damage and inhibits the development of inflammation. Carotenoids also promote the activity of antioxidant enzymes [superoxide dismutase (SOD) and catalase] in the body and strengthen the endogenous antioxidant defense system [121].

Carotenoids modulate the balance of inflammation by promoting the production of anti-inflammatory cytokines (e.g., IL-10) while suppressing the production of inflammatory cytokines [122]. For example, lycopene and lutein have been reported to reduce chronic inflammation by suppressing the expression of pro-inflammatory cytokines IL-1β, IL-6, and TNF-α. This may reduce the progression of chronic diseases such as atherosclerosis and obesity-related diseases. Lutein has been shown to suppress the expression of inflammatory cytokines and reduce atherosclerotic lesions in mouse models of atherosclerosis [123]. Atherosclerosis progresses due to chronic inflammation of the vascular endothelium and is a major cause of CVD. Lutein’s anti-inflammatory action is expected to suppress the inflammation of the vascular endothelium and slow the progression of arteriosclerosis. In animal models of obesity, lycopene was shown to inhibit inflammatory pathways associated with liver fat accumulation [124]. Chronic inflammation associated with obesity is one of the factors that contribute to insulin resistance and non-alcoholic fatty liver disease [124]. Lycopene may reduce the risk of obesity-related diseases by reducing the expression of inflammatory mediators secreted by adipocytes and decreasing fat accumulation in the liver.

### 4.3. Curcumin and Gingerol

As shown in Figure 2G,H, curcumin and gingerol are bioactive compounds derived from plants and have long been used in traditional medicine. These compounds possess potent antioxidant and anti-inflammatory properties and have potential applications in the prevention and treatment of chronic diseases. In particular, by targeting inflammatory signaling pathways, these compounds may contribute to the amelioration of arthritis and chronic inflammatory diseases.

Curcumin is the yellow pigment in “turmeric” and is widely known for its antioxidant and anti-inflammatory properties [125]. Curcumin has traditionally been used in Indian Ayurvedic medicine and Chinese medicine, and in recent years, scientific research has revealed its diverse health benefits. Curcumin inhibits the NF-κB pathway, a major signaling pathway in inflammation, and suppresses the expression of inflammatory cytokines. NF-κB is a transcription factor that promotes the transcription of inflammatory cytokines such as IL-1β, IL-6, and TNF-α, which are involved in the development and progression of chronic inflammation. NF-κB is involved in the onset and progression of chronic inflammation [126]. Curcumin has been shown to reduce inflammatory responses and prevent tissue damage by inhibiting this pathway. Furthermore, curcumin has been reported to have inhibitory effects on cancer cell proliferation through the regulation of the cell cycle and induction of apoptosis (cell death) [127]. For this reason, research is being conducted on both its anti-inflammatory and anti-tumor effects. In animal experiments involving arthritis models, curcumin significantly reduced joint inflammation and alleviated joint pain [128]. Specifically, the administration of curcumin to mice models of arthritis reduced the expression of inflammatory cytokines and suppressed the destruction of joint tissue. Based on this, curcumin is believed to have potential as a therapeutic aid for rheumatoid arthritis and osteoarthritis.

Gingerol is the main bioactive constituent of “Ginger (Zingiber officinale)” and is also the substance responsible for ginger’s distinctive pungent taste. Gingerol has been reported to possess antioxidant and anti-inflammatory properties and is believed to be particularly effective in alleviating arthritis and digestive disorders [129]. Gingerol reduces chronic inflammation by inhibiting the secretion of TNF-α, a pro-inflammatory cytokine that is secreted by immune cells and plays a key role in amplifying inflammation and tissue destruction [130]. By suppressing the secretion of TNF-α, gingerol inhibits the progression of inflammation and contributes to the improvement of inflammation-related diseases. Gingerol has also been reported to reduce pain and swelling by inhibiting the activity of inflammatory enzymes (e.g., cyclooxygenase-2 and induced nitric oxide synthase) and suppressing the production of prostaglandins and nitric oxide [131]. In addition, gingerol suppresses oxidative stress responses and may contribute to the prevention of tissue damage. In mouse models of arthritis, gingerol was shown to inhibit the development of inflammation and reduce the production of inflammatory mediators [132]. Experiments have reported reduced joint inflammation and significantly decreased expression of inflammatory cytokines in mice treated with gingerol. Thus, gingerol is considered useful in relieving symptoms of rheumatoid arthritis and osteoarthritis.

Curcumin and gingerol have the advantage of being readily available as foods and supplements. Curcumin is found as turmeric in curry powder and other products, while gingerol is found in ginger-based foods and beverages. Daily consumption of these compounds may reduce the risk of chronic inflammation and prevent arthritis and other inflammatory diseases. However, curcumin is difficult to dissolve in water and has low bioavailability. For this reason, nanoparticulation and liposome technologies are being studied to enhance curcumin absorption. Gingerol is also being applied to the maintenance of digestive health and the prevention of metabolic diseases. In particular, research on gingerol’s effects on improving digestive function and anti-cancer activity is in progress, and further evidence is expected to be gathered through future clinical trials.

## 5. Molecular Mechanisms

A wide variety of molecular mechanisms are involved in the inhibition of inflammation by plant-derived compounds. In addition to modulating key inflammatory signaling pathways, these compounds regulate chronic inflammation by reducing oxidative stress and suppressing inflammatory gene expression.

### 5.1. Inhibition of the NF-κB Pathway

NF-κB is one of the major transcription factors that regulate the inflammatory response and regulates the expression of many inflammation-related genes, including inflammatory cytokines, chemokines, and adhesion molecules. In chronic inflammation, sustained activation of the NF-κB pathway is observed and plays a central role in many disease progressions. Plant-derived compounds exhibit potent anti-inflammatory effects through inhibition of the NF-κB pathway [133]. First, NF-κB normally exists in the cytoplasm bound to the inhibitory factor IκBα. However, when IκBα is phosphorylated and degraded by inflammatory stimuli (e.g., lipopolysaccharides and TNF-α), NF-κB moves into the nucleus and promotes transcription of inflammatory genes (IL-6, IL-8, TNF-α, etc.) [134]. Polyphenols (quercetin, resveratrol, etc.) inhibit the degradation of IκBα, thereby preventing nuclear transfer of NF-κB and suppressing inflammatory responses [135]. In addition, plant-derived compounds such as chalcone and apigenin directly bind to the p65 subunit of NF-κB and reduce its DNA binding ability, thereby preventing the progression of inflammation [136,137]. Furthermore, ROS are the main factors that activate the NF-κB pathway, and antioxidants such as epigallocatechin gallate eliminate ROS and indirectly inhibit NF-κB activation [138]. These mechanisms have been demonstrated in disease models, with curcumin dramatically reducing inflammatory cytokine production via inhibition of NF-κB in arthritis models and lycopene inhibiting the NF-κB pathway and reducing vascular endothelial inflammation in an atherosclerosis model.

### 5.2. Inhibition of the Inflammasome

The inflammasome is a cytosolic multiprotein complex that plays a pivotal role in the innate immune response by regulating the maturation and secretion of pro-inflammatory cytokines. Among various inflammasome types, the NLRP3 (NOD-like receptor family, pyrin domain-containing 3) inflammasome has been extensively studied due to its involvement in the pathogenesis of numerous chronic inflammatory conditions, including metabolic syndrome, neurodegenerative diseases, and autoimmune disorders [139]. Upon activation by a diverse range of stimuli—such as ROS, extracellular ATP, and crystalline substances—NLRP3 binds to the adaptor protein ASC (apoptosis-associated speck-like protein containing a CARD) and pro-caspase-1, leading to the autocatalytic activation of caspase-1. This, in turn, cleaves pro-IL-1β and pro-IL-18 into their biologically active forms, IL-1β and IL-18, respectively, thereby amplifying the inflammatory response.

Emerging evidence highlights the regulatory role of plant-derived polyphenols and carotenoids in modulating NLRP3 inflammasome activation, offering a promising strategy for inflammation control and chronic disease prevention [140,141]. For instance, resveratrol and quercetin have been shown to attenuate IL-1β and IL-18 secretion by disrupting NLRP3 inflammasome assembly, likely through the inhibition of upstream signaling pathways such as NF-κB and ROS generation. These compounds may also stabilize mitochondrial membranes and preserve redox homeostasis, thereby reducing NLRP3 activation triggers.

Furthermore, luteolin suppresses NLRP3 activity by inhibiting the oligomerization of ASC, which is essential for inflammasome complex formation. This structural interference directly impairs the scaffold necessary for caspase-1 recruitment and activation, thus reducing downstream cytokine maturation. Carotenoids like lutein and lycopene exert similar anti-inflammatory effects through caspase-1 inhibition, which has been linked to decreased IL-1β production in models of metabolic syndrome and atherosclerosis.

These anti-inflammatory mechanisms have been substantiated in various disease models. EGCG, a major catechin in green tea, mitigates insulin resistance in type 2 diabetes models by downregulating NLRP3 inflammasome signaling. Similarly, curcumin has demonstrated neuroprotective properties in Alzheimer’s disease models by attenuating microglial activation and suppressing NLRP3-mediated neuroinflammation [142]. Such findings suggest that targeting the NLRP3 inflammasome with plant-derived compounds holds substantial potential for the development of nutraceuticals and adjunctive therapies for chronic inflammatory diseases.

### 5.3. Control of ROS Generation

Oxidative stress is a major factor that induces and exacerbates inflammation, and excessive production of ROS activates inflammatory signaling pathways. Through their antioxidant activity, plant-derived compounds inhibit ROS production and mitigate the progression of inflammation.

Plant-derived compounds reduce oxidative stress and inhibit the progression of inflammation through the induction of antioxidant enzymes and direct scavenging of ROS [143]. Polyphenols and flavonoids have been reported to reduce oxidative stress by increasing the expression of SOD and converting superoxide anions to hydrogen peroxide, while curcumin enhances SOD activity and inhibits oxidative stress-related inflammation in animal models. Resveratrol is also reported to be an effective antioxidant in the treatment of GPP [144]. Resveratrol also enhances glutathione peroxidase activity and prevents oxidative damage to cells by detoxifying hydrogen peroxide [145]. In addition, lycopene and β-carotene have free radical scavenging properties and protect cell function by preventing lipid oxidation of cell membranes [146]. In disease models, lutein has been shown to inhibit ROS formation in the liver of mice on a high-fat diet model and prevent the progression of fatty liver, while apigenin reduces oxidative stress in neurons and shows neuroprotective effects in a PD model [140]. Thus, plant-derived compounds target diverse molecular pathways and have great potential in the prevention and treatment of chronic diseases.

## 6. Challenges to Practical Application

To maximize the efficacy of plant-derived compounds and promote their practical application, the following issues must be resolved.

### 6.1. Increased Bioavailability

Many plant-derived compounds have been reported to have limited efficacy in the actual human body, although their effects have been clearly confirmed in vitro and in animal models. The main reason for this is low bioavailability. Bioavailability refers to the percentage of ingested compounds that reach the bloodstream and exert their effects on target tissues and cells. Many plant-derived compounds are highly fat-soluble and poorly water-soluble, which limits their absorption from the intestinal tract. Many polyphenols and flavonoids, in particular, may be degraded by intestinal bacteria and enzymes during the digestive process, reducing their activity. In some cases, after a compound is absorbed, its activity is lost due to metabolism in the liver (modification by Phase I and Phase II enzymes). Curcumin, for example, is known to be prone to a decrease in blood levels due to its rapid glucuronide conjugation. Some compounds may not be sufficiently effective against CNS diseases (e.g., AD) because they have difficulty crossing the blood–brain barrier. In the improvement of drug delivery systems, nanocarrier technology and delivery systems utilizing liposomes are attracting attention to improve bioavailability, and encapsulation of compounds in nanoparticles can improve intestinal absorption and control distribution to target tissues. Furthermore, some flavonoids and polyphenols are known to have bioactive metabolites by intestinal bacteria, and their combination with probiotics and prebiotics, which utilize this property to regulate the intestinal microflora, is promising.

### 6.2. Evaluation of Long-Term Safety

Plant-derived compounds are often considered safe because they are “naturally derived”, but comprehensive research on the risks and side effects of long-term ingestion is still limited. Potential risks associated with the safety of plant-derived compounds include accumulation toxicity, interaction problems, and allergic reactions. Some compounds that accumulate in the body with long-term ingestion may cause unexpected toxicity and side effects, particularly high concentrations of polyphenols, which have been reported to reverse antioxidant activity and induce oxidative stress. There is also concern about interaction issues, as these compounds can affect the metabolism of pharmaceuticals by inhibiting or inducing CYP450 enzymes, potentially altering drug efficacy and side effects. In addition, foods and supplements containing multiple polyphenols have been associated with the risk of allergic reactions in some consumers. To mitigate these risks, it is essential to conduct clinical trials, including long-term randomized controlled trials to assess safety, as well as comprehensive toxicity evaluations using animal models and cell studies.

### 6.3. Prospects for Practical Application

To overcome the challenges and effectively utilize plant-derived compounds in medicine and health promotion, the following innovations and new approaches are expected:

Nanocarrier technologies show promise for improving the absorption and target specificity of plant-derived compounds. Advanced drug delivery technologies such as liposomes, nanoemulsions, and polymer-based nanoparticles are being utilized to improve the bioavailability of plant-derived compounds. Liposomes encapsulate compounds in a lipid bilayer to improve absorption and allow efficient uptake from the intestinal tract, and liposomal formulations of curcumin, in particular, have been shown to increase bioavailability several-fold compared to conventional formulations. Nanoemulsions have attracted attention as a technology that converts poorly water-soluble compounds into nano-sized emulsified particles, improving their stability and solubility in the body. In addition, polymer-based nanoparticles using biodegradable polymers (e.g., PLGA) may provide sustained-release drug delivery by encapsulating plant-derived compounds, resulting in sustained efficacy.

### 6.4. Integration into Personalized Nutrition

In recent years, the field of nutrition has focused on “personalized nutrition” based on an individual’s genetic information and gut microbiota profile [147]. Incorporating this approach into the application of plant-derived compounds is expected to bring further benefits. In order to maximize the health benefits of plant-derived compounds, personalized intake planning and digital health technologies are being integrated [147]. In personalized intake planning, a system has been developed to suggest optimal compounds and intakes based on genetic information and the state of the intestinal microflora. In addition, through integration with digital health technology, a system is being built to monitor the effects of the intake of plant-derived compounds in real time using wearable devices and health management applications and is expected to establish optimal intake strategies based on individual health conditions [148].

### 6.5. Plant-Derived Dietary Fiber Resources and Their Effective Utilization

Plant-derived products contain a wide variety of dietary fibers, which are not only important for human health but are useful in the food industry [73,149,150]. In particular, in recent years, dietary fiber contained in inedible parts of fruits and vegetables (peels, seeds, cores, and so on) and edible by-products that tend to be discarded have been reevaluated, attracting attention from the perspective of reducing food loss in the agricultural food supply chain.

For example, apple lees, carrot and beet peels, cabbage leaves, and citrus peels and endosperm are rich in useful dietary fibers such as pectin, cellulose, and hemicellulose [151]. These dietary fibers have been reported to have various health functions, including intestinal regulation, blood sugar and lipid regulation, and even anti-inflammatory effects through the improvement of the intestinal microflora [152]. Furthermore, these by-product-derived fibers are expected to be utilized as functional foods, supplements, and alternative food materials (e.g., gluten-free products and texture modifiers) after powdering or extracting them. From the viewpoint of contributing to the construction of a sustainable food system, effective utilization of these plant-derived fibers is an important issue for future applied research.

## 7. Conclusions

Plant-derived compounds are of great interest in modern medicine and nutrition due to their diverse anti-inflammatory and chronic disease prevention potential. These compounds are abundant in nature and are chemically diverse, including polyphenols, flavonoids, carotenoids, and terpenoids. The potent antioxidant properties they exhibit and their ability to inhibit inflammatory cytokines, block inflammatory pathways, and even regulate the gut microbiota have been shown to play an important role in the prevention and inhibition of the progression of chronic diseases.

The greatest advantage of plant-derived compounds is their ability to approach the multifaceted drivers of chronic disease through their diverse biological effects. By targeting multiple pathways, such as NF-κB and the NLRP3 inflammasome, they exert anti-inflammatory and antioxidant effects, contributing to the prevention of cardiovascular and neurodegenerative diseases. In addition, plant-derived compounds regulate the gut microbiota and may affect mental health via the gut–brain axis, making them a potential complementary therapy for depression and anxiety disorders. The challenges for practical application of plant-derived compounds include improving bioavailability, establishing long-term safety, standardization and quality control, and overcoming legal regulations. Absorption must be improved through the use of nano-carrier technology and liposomes, and side effects and accumulated toxicity must be verified in long-term clinical trials. In addition, standardization must be promoted to prevent variations in quality, and appropriate legislation and scientific rationale must be strengthened for food and pharmaceutical products.

## 8. Future Directions

To advance applied research on plant-derived compounds, it is important to move in the direction of further elucidation of molecular mechanisms, expansion of clinical trials, integration with personalized nutrition, and collaboration with multiple disciplines. First, molecular elucidation of the anti-inflammatory and disease-preventive effects of plant-derived compounds, especially the multi-targeted mechanism of action, will enable more specific applications. However, it is essential to clarify the boundaries of their efficacy, as these compounds do not have the same benefits and individuals do not respond to them equally. Some plant-derived compounds may demonstrate promising effects in vitro or in animal models but fail to show significant clinical benefits in humans due to differences in bioavailability, metabolism, or dosage thresholds.

In addition, since much of the current research is based on animal models and cell studies, human clinical trials must be expanded and improved to confirm actual health effects and improve reliability. These trials should also aim to identify subpopulations that may benefit less—or not at all—from specific compounds, thus preventing overgeneralization of their efficacy. Furthermore, integration with personalized nutrition utilizing genetic information and gut microbiota profiles is expected to develop intake plans for plant-derived compounds that are optimized for individual patients. Additionally, collaboration among experts from multiple disciplines, including medicine, pharmacy, food science, and agriculture, will expand the range of applications of plant-derived compounds and promote their practical use in a wider range of fields.

## Figures and Tables

**Table 1:** Plant-derived bioactive compounds and their rich plant sources.

	High Content Plants	Principal Effect
Quercetin	Apples, onion	Antioxidant, anti-inflammatory effect
Epigallocatechin gallate	Green tea leaves	Antioxidant, anti-inflammatory effect
Luteolin	Celery, bell peppers	Antioxidant, anti-inflammatory effect
β-carotene	Carrots, squash	Antioxidant, anti-inflammatory effect
Lycopene	Tomatoes, watermelon	Anti-inflammatory, and reduction the risk of obesity-related diseases effect
Lutein	Spinach, kale	Anti-inflammatory effect
Curcumin	Curcuma longa	Antioxidant, anti-inflammatory effect
Gingerol	Zingiber officinale	Anti-inflammatory effect

## Data Availability

The datasets used and/or analyzed during this study are available from the corresponding author upon reasonable request.

## Abbreviations

The following abbreviations are used in this manuscript:

CVD	cardiovascular disease
IL-1β	interleukin-1β
IL-6	interleukin-6
TNF-α	tumor necrosis factor-α
ROS	reactive oxygen species
NSAIDs	nonsteroidal anti-inflammatory drugs
NF-κB	nuclear factor-κB
MAPK	mitogen-activated protein kinase
AD	Alzheimer’s disease
PD	Parkinson’s disease
CNS	central nervous system
SOD	superoxide dismutase
